# Parallel multiple instance learning for extremely large histopathology image analysis

**DOI:** 10.1186/s12859-017-1768-8

**Published:** 2017-08-03

**Authors:** Yan Xu, Yeshu Li, Zhengyang Shen, Ziwei Wu, Teng Gao, Yubo Fan, Maode Lai, Eric I-Chao Chang

**Affiliations:** 1 State Key Laboratory of Software Development Environment and Key Laboratory of Biomechanics and Mechanobiology of Ministry of Education and Research Institute of Beihang University in Shenzhen, Beijing, China; 2 Microsoft Research Asia, Beijing, China; 3 School of Computer Science and Engineering, Beihang University, Beijing, China; 4 Department of Pathology, School of Medicine, Zhejiang University, Zhejiang, China; 5 College of Information and Electrical Engineering, China Agricultural University, Beijing, China

**Keywords:** Histopathology image analysis, Microscopic image analysis, Multiple instance learning, Parallelization

## Abstract

**Background:**

Histopathology images are critical for medical diagnosis, e.g., cancer and its treatment. A standard histopathology slice can be easily scanned at a high resolution of, say, 200,000×200,000 pixels. These high resolution images can make most existing imaging processing tools infeasible or less effective when operated on a single machine with limited memory, disk space and computing power.

**Results:**

In this paper, we propose an algorithm tackling this new emerging “big data” problem utilizing parallel computing on High-Performance-Computing (HPC) clusters. Experimental results on a large-scale data set (1318 images at a scale of 10 billion pixels each) demonstrate the efficiency and effectiveness of the proposed algorithm for low-latency real-time applications.

**Conclusions:**

The framework proposed an effective and efficient system for extremely large histopathology image analysis. It is based on the multiple instance learning formulation for weakly-supervised learning for image classification, segmentation and clustering. When a max-margin concept is adopted for different clusters, we obtain further improvement in clustering performance.

## Background

Histopathology provides some of the most critical information for cancer diagnosis [1]. By analyzing the histopathology images of a patient, we can predict presence or absence of cancer for a patient probabilistically to support the pathologist in making a proper analysis. The whole-slide images with high resolution are helpful for pathologists to conduct researches on cancer subtypes [2]. The digitized information also makes the approaches and analysis more quantitative, objective and tenable. With the help of ever-increasing computer resources and related computer software, automated analysis of histopathology images really help pathologists make faster and more accurate diagnosis [3].

However, extremely large histopathology images with enormous amounts of pixels create a bottleneck for applying traditional Computer Aided Diagnosis (CAD) tools [3], which often operate on a single machine with limited memory and space. In our data set, for example, a digitized histopathological image with a resolution of 226 nm per pixel can have a size of 148,277×156,661 pixels. It is common that pathological section processing generates 12-20 images for each patient [1]. Even if we use only 12 images generated by just one patient in the training stage, which is rarely the case in reality, with a traditional method, it will take 65 GB of memory to load a whole single image once in a computer and approximately 100 h to train on a single core of a Quad-core Xeon 2.43 GHz processor according to our experiment results. However, a quick response is usually required in clinical practice, especially in the frozen section procedure, in which the pathologist has to make a therapeutic decision and tell the surgeon in fewer than 15 min [4] after cryosection images are received. Regardless of whether there is enough storage space in a normal PC, it will take tens of hours, out of scope in a cryosection decision stage, to process one patient’s slices in the data distribution stage, the feature extraction stage and the prediction stage with a single core mentioned above. Therefore, it is infeasible to handle such large images with a single computer. In order to address the problem, a learning method, whose processing time is viable for clinical practice, is desired.

Weakly supervised learning, more specifically Multiple Instance Learning (MIL) [5], fits into the analysis for histopathology cancer images because it uses coarse-grained labeling to aid automatic exploration of fine-grained information. In a whole-slide image, there are lots of pieces randomly cropped, called bags in this paper. Patches, or instances, consisting of pixels, are sampled from each piece. So we have three different levels of classifiers, image-level, instance-level and pixel-level classifiers. The advantage brought by MIL for histopathology analysis is that if an instance-level classifier is trained, automatic pixel-level segmentation (cancer vs. non-cancer regions) could be performed. Image-level classifier could also be directly obtained under the MIL setting and then achieve image-level classification (cancerous or non-cancerous). Moreover, in histopathology image analysis, it is desirable to discover the subclasses of various cancer tissue types to help pathologists make better diagnosis. As a general protocol for cancer subtype classification is not all available, patch-level clustering (different cancer subtypes) of cancer tissues is noticed by researchers. Xu et al. embed the clustering concept into the MIL setting, proposing the Multiple Clustered Instance Learning (MCIL) [6] method based on MIL and under the boosting framework, which is able to perform image-level classification, pixel-level segmentation and patch-level clustering altogether for histopathology images. The pathologist can use the classification results to reasonably analyze whether there is cancer or not for a patient. The segmentation results could be used to discover cancerous regions. Furthermore, the prognosis of the patient could be judged by the clustering results of cancer subtypes. However, training those models such as MCIL on large data sets is extremely computationally intensive. Additionally, the performances of MCIL seriously depend upon initialization of cancer subtypes through a single clustering process, resulting in poorly alignment of clusters and thus limited discriminative properties of cancer subtypes. Though the performances of MCIL in classification and clustering are already relatively high, it fails in segmentation tasks.

**Fig. 1:**
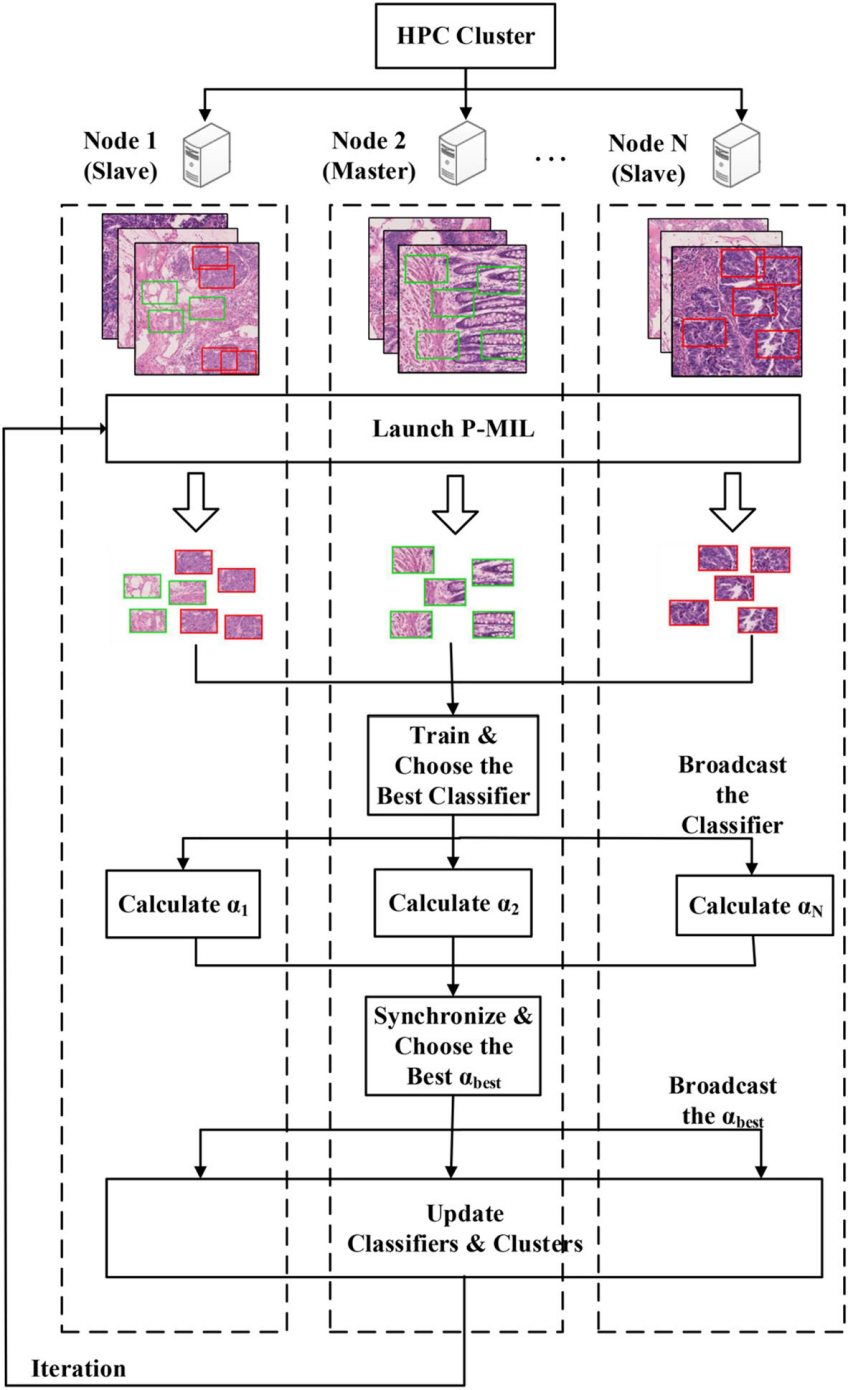
Parallel Multiple Instance Learning (P-MIL) on High-Performance-Computing (HPC) cluster. Red: positive instances; Green: negative instances. At first, we divide and distribute data to the nodes. The master will collect the results calculated by individual nodes, train multiple classifiers and choose the best one. Next, the slaves receive the best weak classifier and calculate an individual *α* value. The master node then will synchronize all the nodes, choose the *α*_*best*_ and broadcast it. At last, all the nodes will update classifiers with the *α*_*best*_ and update new clusters with the new classifiers through communication, in which the master will coordinate to ensure data coherence. The program will continue running in a loop until the loop ends

In this paper, we have developed a Parallel Multiple Instance Learning (P-MIL) algorithm on High-Performance-Computing (HPC) clusters, using a combination of Message Passing Interface (MPI) [7] and multi-threading [8]. The algorithm parallelizes a multiple instance learning strategy and is implemented based on the hybrid MPI/multi-threading programming model. We also introduce a max-margin approach to intensifying competition among clusters in our P-MIL method. By applying the max-margin concept, the discriminative ability of our classifiers and the purity of our clustering results benefit each other. In addition, we conduct a thorough experiment study in which our model is trained by millions of instances, each with feature vectors of 215 dimensions, in 128 compute nodes (1024 CPU cores) for 11.6 h successfully. We offer the experimental results as well as analysis in support of our method. Our experiments are conducted on a Microsoft Windows HPC [9, 10] cluster, which is a homogeneous infrastructure consisting of 128 compute nodes, connected by network with high bandwidth and low latency. Each compute node has 2 Quad-core Xeon 2.43 GHz processors, 16 GB Random Access Memory (RAM), 1 Gbps Ethernet adapters and 1.7 TB local disk storage. The prediction time for images generated by one patient with our method is about 382.79 s. So the short processing time makes our work applicable in clinical practice. P-MIL is also a general model, capable of being applied to medical image analysis as well as many other domains. Figure 1 is the flow diagram for P-MIL.

Our approach also differs from existing formulations in machine learning in the following aspects: In MCIL, cancer subtypes are initialized through clustering and fixed in the learning phase. The corresponding strong classifiers are updated individually through boosting. Although MCIL introduces clustering, it assumes no max-margin concept among clusters [6]. Other than solely updating classifiers, a sort of clustering competition mechanism is introduced in this paper to optimize clusters simultaneously, representing latent cancer subtypes. By combining these two operations, distributions of clusters as well as discriminative abilities of corresponding classifiers can be improved to achieve better comprehensive performance as shown in our experimental results. Context-constrained Multiple Instance Learning (ccMIL), proposed by Xu et al. [11] as well, emphasizes the segmentation task using the contextual information of instances as a prior. Above all, none of the above methods except for P-MIL are targeted for large scale data and their processing times make them not applicable in clinical practice.

## Related work

Medical image analysis, including 2D and 3D medical images, has been a popular and active research field for many years. There are also some works about histopathology image analysis. In 1999, Adiga et al. [12] introduced a watershed algorithm as well as a rule-based merging technique into their method to work out segmentation of 3D histopathology images. In 2009, Caicedo et al. [13] adopted bag of features and kernel functions for Support Vector Machine (SVM) to deal with histopathology image classification tasks. In the same year, Gurcan et al. [3] summarized the development and application of histopathology image analysis, especially for CAD technology. In 2011, Lu et al. [14] proposed a technique with radial line scanning, aimed at detecting melanocytes from keratinocytes in skin histopathology images. Two years later, an automated technique was put forward by Lu et al. [15] to perform segmentation and classification on whole slide histopathological images with 90% classification accuracy. In 2016, Barker et al. [16] came up with an automated classification approach to classifying pathology images by brain tumor type, with the help of localized characteristics in images.

Because of inherent ambiguity, time-consuming work and difficulties with manual labeling, the Multiple Instance Learning methods succeed in digging fine-grained information from coarse-grained information so that the burden of manpower for labeling could be eased. Fung et al. [17] adopt the Multiple Instance Learning method and improve it to deal with problems in processing medical images in application of CAD, which pays close attention to medical diagnosis. A new approach for categorization is proposed by Bi et al. [18] to search for pulmonary embolism from some images. Two novel formulations which extend Support Vector Machines (SVMs), presented by Andrews et al. [19], achieve good results when applied to the MUSK [20] data sets, the benchmark data sets. Babenko et al. [21] even use on-line Multiple Instance Learning to deal with object tracking problems. Nguyen et al. [22] propose an active-learning method to classify medical images. Chen et al. [23] put forward a multi-class multi-instance boosting method to detect human body parts in image processing. Qi et al. [24] integrate MIL into SVM to perform image annotations automatically. Therefore, the MIL framework can be applied to a lot of domains, especially medical image analysis. Due to the characteristics of histopathology images, it is suitable to apply MIL models to process the images.

There have been some works about Multiple Instance Clustering before, which is a method for clustering in MIL problems. Zhang et al. [25] develop a kind of Multiple Instance Clustering method to partition bags of instances about images into different clusters. They combine the Multiple Instance Clustering method with the Multiple Instance Prediction method to solve the unsupervised Multiple Instance Learning problem. Xu et al. [26] also develop a max-margin clustering method to find max-margin hyperplanes among data and to label the data in a wider sense. Furthermore, a model, which considers relations among data and produces coherent clusters of data, is proposed by Taskar et al. [27] to extend the Multiple Instance Learning method into wider domains to deal with more real-world problems. A novel Multiple Instance Clustering as well as prediction is proposed by Zhang et al. [28] to tackle the unsupervised MIL task.

However, the aforementioned works as well as works about processing histopathology images mostly focus on a small data set of small images. For instance, Xu et al. [29] experiment on a data set of 60 histopathology images, including stained prostate biopsy samples and whole-mount histological sections. Doyle et al. [30] conduct experiments on a data set of 48 histopathology images of breast biopsy tissue even though they focus on complex features. Furthermore, tens of histopathology images are used for the experiments in [31] for segmentation. In [32], fewer than 100 histopathology images, consisting of digital images of breast biopsy tissue, are used for experiments of classification. The works mentioned above about histopathology images are dealing with a small number of small images. So they may not be applicable in face of problems with large-scale data sets, for example 3.78 TB of data in our experiments, or in practical application.

Since the idea of “big data” came out recently, it is inevitable that medical images are involved as well. A lot of researchers have already noticed the “big data” problem that medical image analysis faces. In [33], the authors indicate that with increased amount of medical image data Content Based Image Retrieval (CBIR) techniques are required to process large-scale medical images more efficiently. Latent Semantic Analysis (LSA) is applied to large-scale medical image databases in their work. Kye et al. [34] propose a GPU-based Maximum Intensity Projection (MIP) method with their visibility culling method to process as well as illustrate images at an interactive-level rate. In their experiments, every single scan can generate more than one thousand images for a patient. It is suggested in [35] that the exponential increase in biomedical data requires more efficient methods to be proposed to tackle problems close to real-world problems. Moreover, Huang et al. [36] put forward a platform, including GPU-based sparse coding and dynamic sampling techniques, to speed up analysis of histopathological whole slide images, which can take hundreds of hours to process a whole set of whole slide images high power fields originally. A novel framework based on point set morphological filtering is proposed in [37] to process large-scale histopathological images as well.

There are a few existing works about parallel or distributed algorithms for medical image analysis. The most related work is that of Aji’s [38]. Aji et al. propose a spatial query framework for large scale pathology images based on MapReduce. The framework is evaluated by 10 physical nodes and 192 cores (AMD 6172, 2.1 GHz) on Cloudera Hadoop-0.20.2-cdh3u2. The experiment shows that the framework can support scalable and high performance spatial queries with high efficiency and scalability. Pope et al. [39] simulate a realistic physiological multi-scale model of heart using hybrid programming models. In 2017, Wei et al. [40] map MIL bags to vectors for better scalability.

Other than medical image analysis, there are a lot more works about parallel algorithms. In machine learning, Xiao [41] conducts a survey about parallel and distributed computing algorithms. These algorithms include K-Nearest Neighbor (KNN), Decision tree, Naive Bayes, K-means, Expectation-Maximization, PageRank, Support Vector Machine, Latent Dirichlet Allocation, and Conditional Random Fields [41]. Srivastava et al. [42] propose a parallel formulation of their serial algorithm about classifiers for data mining. Aparicio et al. [43] propose a parallel implementation of the KNN classifiers to tackle large-scale data mining problems. Zeng et al. [44] propose a hybrid model of MPI and Open Multi-Processing (OpenMP) to deal with the communication work during parallelization, which considers both running efficiency and code complexity. In [45], Pacheco et al. make a detailed description about programming with MPI parallelization concepts. A novel iterative parallel approach dealing with unstructured problems about linear systems is proposed by Censor et al. [46]. In addition, Zaki et al. [47] come up with a parallel classification method used for data mining. Moreover, He et al. [48] propose a parallel extreme SVM algorithm based on MapReduce, that is able to meet the need of tackling big-data problems and on-line problems. A software system, which could distribute image analysis tasks to a distributed and parallel cluster with many compute nodes, is developed by Foran et al. [49]. Thus parallel methods are alike to some degree, most of which are aimed at distributing computing tasks to different compute nodes to make full use of the computing ability of the nodes. Moreover, many experimental results show that a hybrid parallelization model is better than a model using only one sort of parallelization technique. That’s why we come up with a hybrid model of multi-threading and MPI to help implement the parallel framework for the MIL method. No previous work has ever applied a parallelized method to dealing with histopathology image analysis in practical application.

It is worth mentioning the history of our research work because it makes a clear and logical path from the origin to our current work. At first, we develop MCIL and ccMIL but both of them were merely applied to relatively small-scale images. Facing the demand of clinical practice and expecting a method applicable in many organs, we have to develop the P-MIL method. Unlike the P-MIL method, previous works such as MIL [50], MCIL [6] and ccMIL [11] mainly focus on the process of learning a classifier to enhance accuracy, though infeasible in clinical application. As mentioned before, P-MIL mainly contributes a parallelized algorithm to make it applicable in real scenes and a max-margin concept about competition among clusters to further improve accuracy of classifiers. The whole process of the project includes the full guidance of pathologists. Apart from the colon histopathology images we use, hospitals are collecting brain tumor images and gastric carcinoma images as well.

## Methods

P-MIL is a parallelized multiple instance learning formulation and able to maximize margin among clusters. It is based on MIL and under the boosting framework, meanwhile, taking patch-level clustering into consideration. The basic framework of our P-MIL method is able to perform classification, segmentation and clustering altogether. Our P-MIL framework introduces a max-margin concept to enhance the competition among clusters thus achieves better overall performance. With the development of cluster computing, parallel algorithms make a lot of sense in reality. The parallelized structure of our P-MIL method effectively shortens the execution time, which makes it possible for practical application.

In this section, first, we overview the basic MIL framework of our parallel algorithm. Second, we show our max-margin concept on competition of clusters. Finally, we introduce our parallel computing techniques, MPI and multi-threading. Additionally, we present a detailed pseudo code for P-MIL.

### Multiple instance learning framework for classification, segmentation, and clustering

Fully supervised approaches for histopathology image analysis require detailed manual annotations, which are not only time-consuming but also intrinsically ambiguous, even for well-trained experts. Standard unsupervised approaches usually fail due to their complicated patterns. The MIL framework works well for the task because it takes advantage of both supervised approaches and unsupervised approaches.

In our framework, the cancer and non-cancer pieces, randomly cropped from the whole histopathology slices (called images in this paper), are considered as positive and negative bags respectively. The patches densely sampled from these pieces are considered as instances. In the MIL framework, a bag is labeled as positive if at least one of the instances in the bag is considered as positive. In other words, if we find cancer cells in a small patch, the patient is regarded as a cancerous patient.

We assume that *x*_*i*_ represents the *i* ^*th*^ bag in training data $\mathcal {X}$: $x_{i} \in {\mathcal {X}}=\{x_{1}, \dots, x_{n}\}$ (n is the number of bags). For each bag, $y_{i} \in {\mathcal {Y}}=\{-1, +1\}$ is the corresponding label for *x*_*i*_. +1 represents positive while -1 represents negative.

*x*_*i*_={*x*_*i*1_,…,*x*_*im*_}, consisting of *m* instances (*m* is the number of instances in the *i* ^*th*^ bag). Histopathology cancer images include multiple types of instances, each of which belongs to one of the clusters, denoting cancer subtypes or non-cancer. Initially, the clustering operation divides the instances into *K* clusters of positive instances and a negative instance cluster. For each instance and a sort of positive cluster, there is a latent variable: $y_{ij}^{k}\in {\mathcal {Y}}=\{-1, +1\}$, denoting whether the instance *x*_*ij*_ belongs to the *k* ^*th*^ positive cluster, where *k*∈{1,…,*K*}. *j*, which varies from 1 to *m*, represents the label of an instance with regard to a specific bag. *i* represents the corresponding bag. Here, *y*_*i*_ and $y_{ij}^{k}$ have the same value range. A bag is labeled as positive if at least one of its instances belongs to at least one of the *K* clusters:

1 $$ y_{i} = \max_{j}\; \max_{k}{\left(y_{ij}^{k}\right)}.  $$

**H**(*x*_*i*_) and **h** ^*k*^(*x*_*ij*_) are a bag-level classifier and an instance-level classifier respectively, which are to be learned in the method later, where

2 $$ \mathbf{H}(x_{i}) = \max\limits_{k}\max\limits_{j}{\mathbf{h}^{k}(x_{ij})}.  $$

The training data consists of $\mathcal {X}$ and $\mathcal {Y}$. **h** ^*k*^ represents the *k* ^*th*^ instance-level classifier for the *k* ^*th*^ cancer subtype.

The Multiple Instance Learning-Boost (MIL-Boost) [50] framework is employed to instantiate the approach in this paper. The loss function we choose is defined in the AnyBoost method [51] :

3 $$ \begin{aligned} \mathcal{L}(\mathbf{h}) &= -\sum_{i=1}^{n}w_{i}\left({\mathbf{1}}\left(y_{i}=1\right)\log{p_{i}}+{\mathbf{1}}\left(y_{i}=-1\right)\right.\\ &\quad\left.\times\log{(1-p_{i})}\right) \end{aligned}  $$

4 $$ \hspace{14pt} p_{i}\equiv p\left(y_{i}=1|x_{i}\right),  $$

where 1(·) is an indicator function, *p*_*i*_ is a function of **h** and $\mathcal {L}(\mathbf {h})$ is a function of *p*_*i*_ at a bag-level. The loss function is the standard negative log likelihood. *w*_*i*_ is the prior weight of the *i* ^*th*^ training data. The probability *p*_*ij*_ of an instance *x*_*ij*_ is:

5 $$ p_{ij}=\sigma\left(2\mathbf{h}_{ij}\right), \qquad \mathbf{h}_{ij}=\mathbf{h}(x_{ij}).  $$

The probability *p*_*i*_ is the maximum in *p*_*ij*_.

For differentiation purposes, a soft-max function [52], a differentiable approximation of max, is then introduced. For a set of *m* variables, **v**={*v*_1_,*v*_2_,…,*v*_*m*_}, the soft-max function *g*_*l*_(*v*_*l*_) is defined as:

6 $$ \begin{aligned} g_{l}(v_{l}) &\approx \max\limits_{l}(v_{l}) = v^{*},\\ \frac{\partial g_{l}(v_{l})}{\partial v_{i}} &\approx \frac{{\mathbf{1}}\left(v_{i} = v^{*}\right)}{\sum\limits_{l} {\mathbf{1}}(v_{l} = v^{*})}, m = |\mathbf{v}|. \end{aligned}  $$

Using the soft-max function *g* in place of the max function, we can write *p*_*i*_ as:

7 $$ p_{i} = g_{j}\left(g_{k}\left(p_{ij}^{k}\right)\right) = g_{jk}{\left(p_{ij}^{k}\right)} =g_{jk}\left(\sigma\left(2\mathbf{h}_{ij}^{k}\right)\right)  $$

8 $$ \sigma(v) = \frac{1}{1+\exp{(-v)}}, \qquad \mathbf{h}_{ij}^{k} = \mathbf{h}^{k}(x_{ij}).  $$

The function $g_{jk}\left (p_{ij}^{k}\right)$ could be understood as a function *g* including all $p_{ij}^{k}$ indexed by *k* and *j*. In this paper, the generalized mean (GM) model [53] is chosen as the soft-max function.

We can train the weak classifier $\mathbf {h}_{t}^{k}$, where *t* denotes the *t* ^*th*^ round iteration, by using the weight $|w_{ij}^{k}|$ to find the minimum error rate. The weight $w_{ij}^{k}$ can be written as

9 $$ w_{ij}^{k} = -\frac{\partial{\mathcal{L}(\mathbf{h})}}{\partial{\mathbf{h}_{ij}^{k}}} = -\frac{\partial{\mathcal{L}(\mathbf{h})}}{\partial{p_{i}}} \frac{\partial{p_{i}}}{\partial{p_{ij}^{k}}}\frac{\partial{p_{ij}^{k}}}{\partial{\mathbf{h}_{ij}^{k}}}.  $$

Here,

10 $$ \frac{\partial{\mathcal{L}(\mathbf{h})}}{\partial{p_{i}}} = \left\{ \begin{array}{ll} -\frac {1}{p_{i}} & if \ y_{i} = 1 \\ \frac{1}{1-p_{i}} & if \ y_{i} = -1 \end{array} \right.  $$

11 $$ \frac{\partial{p_{i}}}{\partial{p_{ij}^{k}}} = p_{i} \frac{\left(p_{ij}^{k}\right)^{r-1}}{{\sum\nolimits}_{j,k}\left(p_{ij}^{k}\right)^{r}}, \qquad \frac{\partial{p_{ij}^{k}}}{\partial{\mathbf{h}_{ij}^{k}}} = 2{p_{ij}^{k}}\left(1-{p_{ij}^{k}}\right).  $$

Finally, we get a strong classifier **h** ^*k*^:

12 $$ {\mathbf{h}}^{k} \gets {{\mathbf{h}}^{k}+\alpha_{t}^{k} \mathbf{h}_{t}^{k}},  $$

13 $$ \begin{aligned} \mathbf{h}_{t}^{k} &= arg\,min_{\mathbf{h}}\sum_{ij}{{\bf{1}}\left(\mathbf{h}\left(x_{ij}^{k}\right)\neq y_{i}\right)|w_{ij}^{k}|},\\ \alpha_{t}^{k} &= arg\,min_{\alpha}\mathcal{L}\left({{\mathbf{h}}^{k}+\alpha \mathbf{h}_{t}^{k}}\right). \end{aligned}  $$

$\mathbf {h}_{t}^{k}$ is chosen from the weak classifiers trained with feature histograms, and $\alpha _{t}^{k}$ is chosen by using a line search method.

For training, we have to choose a kind of appropriate weak classifier. The only requirement for a weak classifier or a weak learner is that it is better than random guessing [54], so that’s why weak classifiers are always simple and easy to build. By applying boosting to weak classifiers, they can be trained and combined to be strong classifiers.

A decision stump [55] is a special decision tree consisting of a single level. As a weak classifier in a machine learning model, a decision stump is a desirable base learner for ensemble techniques. A full decision tree is accurate but time-consuming. In consideration of the efficiency of the algorithm and the implementation of parallelization, we adopt a previously proposed weak classifier, which could be called multi-decision stumps [50]. It is a combined classifier with multiple thresholds to be trained. Achieving high accuracy as well as high efficiency, the multi-decision stump classifier performs well in experiments.

We use a boosting framework for training, learning and updating classifiers. For each iteration step, each cancer subtype and each instance, we calculate the weight $|w_{ij}^{k}|$ at first. Then we have a weighted histogram for each feature in this instance. Classifiers are trained based on the generated weighted histograms [56], one for each feature [57]. Lastly, the best classifier with the minimum error rate is chosen to be the best weak classifier. With this classifier, we use a line search method to find the best $\alpha _{t}^{k}$ to minimize the loss function value. A strong classifier is updated afterwards. Boosting is adopted and instantiated in our approach in that it is also compatible to parallelism.

### Max-margin concept

**Fig. 2:**
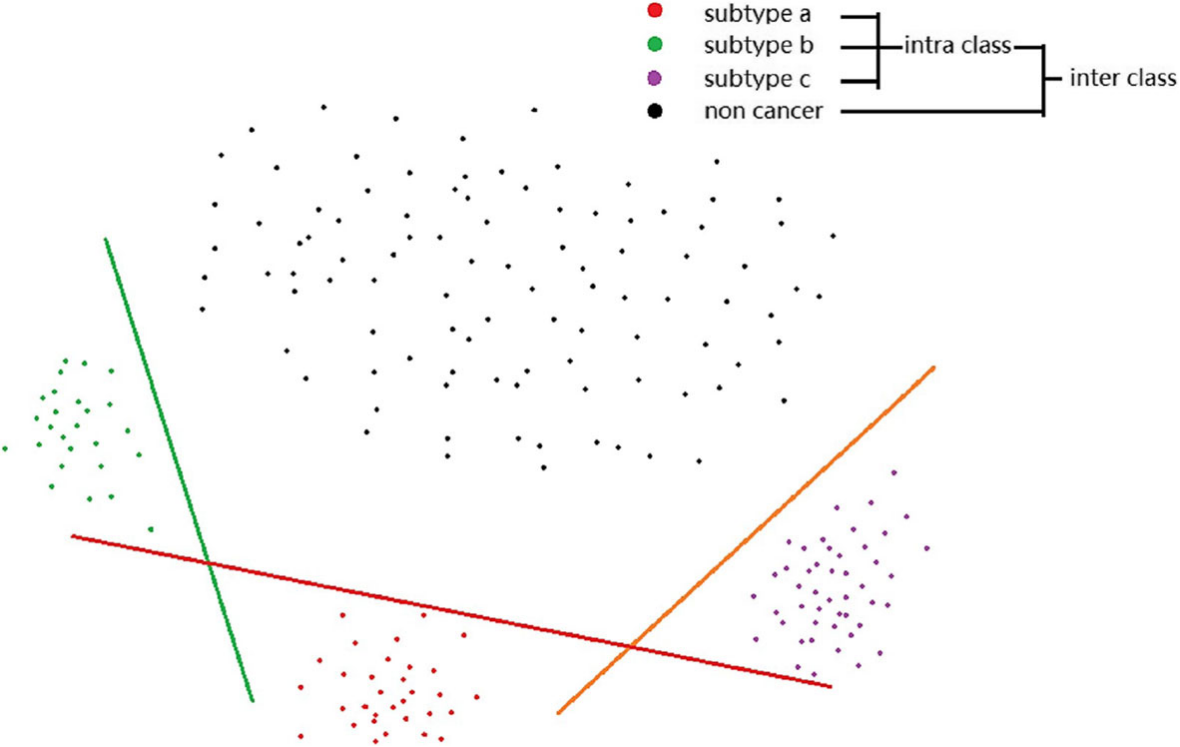
Illustrations of max-margin using linear classifier. *Green*, *red* and *purple* dots represent three specific cancer subtypes, while *black* dots represent non-cancer instances. Linear boundaries are trained to separate cancer subtypes from each other (intra-class) and the non-cancer (inter-class)

The margin between two clusters is defined as the minimum distance between the hyperplane for the two clusters and any data point belonging to the two clusters. Margin is determined by classifiers, whose reliability indicates accuracy and clarity of clustering. A max-margin algorithm is aimed to maximize the aforementioned distance, more specifically, the difference between the true category label of the sample and the best runner-up [58]. In this paper, we conduct classifiers training and cluster competition simultaneously to realize max-margin. Specifically, cluster competition maximizes the intraclass difference (cancer subtype vs cancer subtype), which is one of the characteristics of the cancer images, and greatly accelerates the convergence of the boosting algorithm. At the same time, the boosting framework learns discriminative classifiers for both intra-classes and inter-classes (cancer subtype to non-cancer). Figure 2 illustrates the max-margin concept by using linear classifier.

**Fig. 3:**
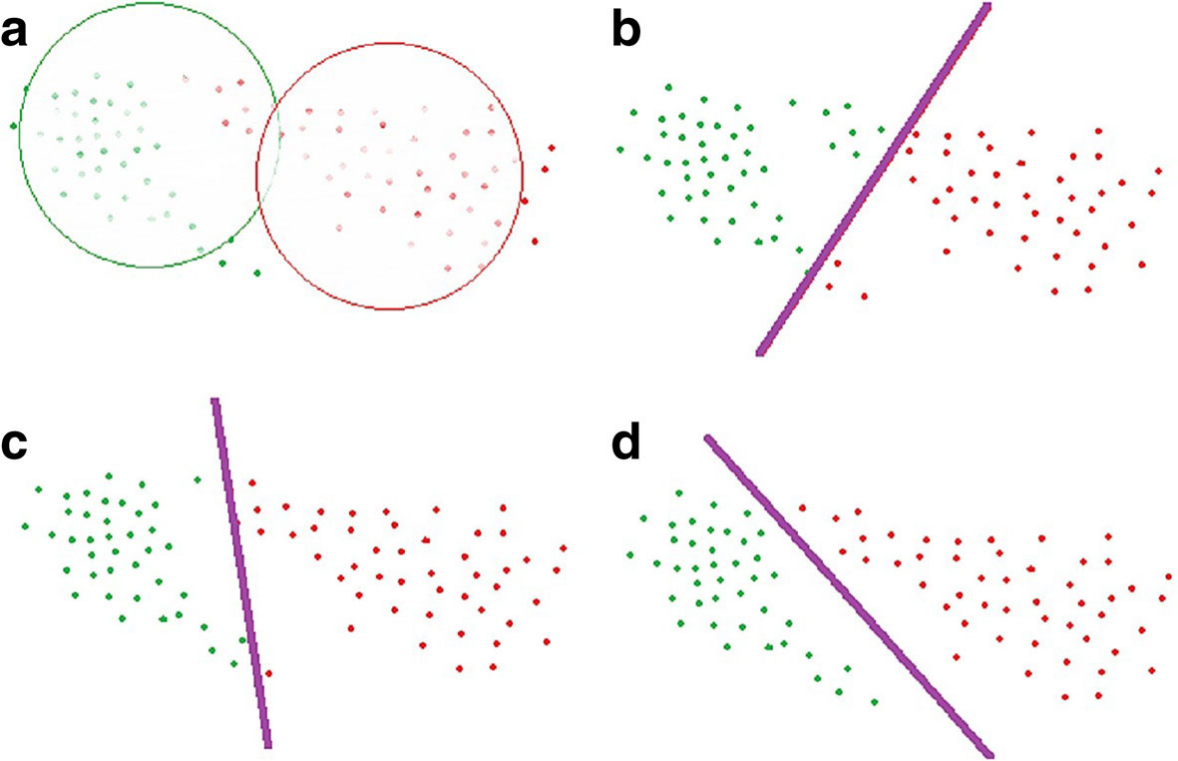
Illustrations of cluster competition using max-margin linear classifier. *Green* and *red* dots represent two classes. In **a**, two classes are initialized by K-means method. In **b**–**d**, cluster competition takes place until the model converges. Specifically, instances in each class are classified by linear classifiers, according to which they update their labels. Then, a new classifier is trained based on the new labels. The cluster competition converges when both classifiers and labels of instances become in a stable state

Due to lack of explicit competition among clusters, MCIL [6] is not well aligned for clusters. In this paper, we explicitly maximize margin in clustering. To achieve this goal, in the initial stage, we use *K*-means [59] algorithm to divide all the positive instances into *K* clusters, where the positive instance sets are $D_{1}^{+}=\left \{D_{1}^{1}, D_{1}^{2}, \dots, D_{1}^{K}\right \}$ and the negative instance set is $D_{1}^{-}$. When in the *t* ^*th*^ iteration, for training a weak classifier $\mathbf {h}_{t}^{k}$, we choose the positive training data as $D_{t}^{k}$ and the negative training data as $\left (D_{t}^{+} - D_{t}^{k}\right) \bigcup D_{t}^{-}$ instead of just $D_{t}^{-}$. The $\mathbf {h}_{t}^{k}$ would then concatenate to **h** ^*k*^ as a step of the boosting framework. Afterwards, instead of making the instances in clusters fixed all the time, we update the cluster label of every instance at the end of each iteration. Specifically, after *t* iterations of training, we use the trained classifier to compute $p_{ij}^{k}$ and to generate new sets of positive instances, $D_{t+1}^{+}=\left \{D_{t+1}^{1}, D_{t+1}^{2}, \dots, D_{t+1}^{K}\right \}$. Figure 3 illustrates a simple update process of two clusters using linear classifier.

Upon updating, the instance *x*_*ij*_ belongs to the *k* ^*th*^ cluster, so that it is classified with the highest probability by the *k* ^*th*^ strong classifier **h** ^*k*^. In this way, the updated division of the training instances maximizes the differences among the clusters and indicates the most discriminative ability of the current cluster classifiers, resulting in strong competition.

For some novel but small clusters, when competing with bigger clusters, they tend to be dying out if the margin is too small to distinguish the clusters. So the max-margin method could effectively reduce the possibility of the aforementioned situation as much as possible. For example, it is impossible for a pathologist to remember all the cancer subtypes. Furthermore, some rare subtypes may have only a few instances available for training. The max-margin concept is introduced to enhance competition thus distinguishing the rare subtypes from others, which can make prognosis much easier.

### Parallel multiple instance learning

#### Parallel programming models

In our work, we utilize both MPI and multi-threading techniques to implement parallelization. All that we want to do is to parallelize our algorithm, and MPI is just a convenient tool for parallel implementation. Multi-threading is a widespread parallel programming and execution model that aims to maximize utilization of multi-core processor computers. Data sharing across different nodes in HPC cluster could be done by cross-process communication. We adopt MPI where data sharing is done by one process sending data to other processes.

Although the MPI parallel programming model could already enable application to scale up in HPC cluster, previous studies [39, 60] show that a hybrid model has more advantages. The MPI/multi-threading hybrid parallel model is a combination of MPI as inter-node communication and multi-threading as intra-node parallelism. It uses only one process per node for MPI communication calls, thereby reducing memory footprints, MPI runtime overhead and communication traffic. Each MPI process is consisting of several threads, one of which as the master thread for inter-node communication and all of which could be assigned computation work.

The MIL algorithm has the data parallel nature that the most compute-intensive tasks can be divided and executed simultaneously and independently. Since every image bag can be treated independently before every synchronization stage, the prior weight for each training data bag, the weighted histograms for instances, the loss function values for choosing *α*_*best*_ and the updating behaviors for clusters with refreshed classifiers can all be done in parallel. After distributing and dispatching the tasks, a simple synchronization step will bring the algorithm procedure back to normal un-parallel routine.

Considering the architecture of the HPC cluster and the data parallel nature of the MIL algorithm, we adopt this hybrid parallel model, which is highly parallelized and achieve satisfactory performance.

#### Implementation of P-MIL

We parallelize the MIL by utilizing its data parallel nature and implement it in two stages: the data distribution stage and the MIL training & searching stage.

In the data distribution stage, we partition the large-scale data set $\mathcal {X}$ into multiple disjoint data subsets, and distribute them evenly to HPC cluster nodes. Other input data is so small that every node can have a copy of it. We use an image bag as a unit for data partition and distribution, so in the next stage the values of the instances belonging to the same bag could avoid being exchanged across different nodes, which saves a lot of communication cost.

In the training & searching stage, we use the hybrid parallel model in which each node will work independently calculating on data subsets cached in its local disk or memory by multi-threads, and do inter-node communication through MPI to exchange partial results.

For inter-node collaboration, we use the master-slave paradigm to implement it. Among all the nodes on HPC, we assign one node as the master node, and others as slaves (actually, we reuse one slave node to launch a master process because master codes and slave codes have no computational overlap). The master node is mainly responsible for global-level sequential operations, such as choosing the best $\mathbf {h}_{t}^{k}$ and updating **h** ^*k*^. The master is the core of communication and synchronization, controlling the whole parallel program. For example, determining the best weak classifier, choosing the best $\alpha _{t}^{k}$ to minimize the loss function value, distributing the determined value to other nodes and dispatching data-transfer tasks to the querying nodes are some of the responsibilities of the master in P-MIL. The slaves are the actual computational nodes running compute-intensive code based on its data subsets, such as computing $w_{ij}^{k}$ and computing histogram of $x_{ij}^{d}$. As mentioned before, among master and slaves, we use MPI for their communication. On each slave node, we use multi-threading to do intra-node parallelism. Each slave node launches one process consisting of eight Windows threads, each on a core. The eight threads work independently on disjoint image bags and update shared values (such as histogram of $x_{ij}^{d}$) in memory with protection by critical section. The computation work of each thread has no influence on the computation work of others. That is the main idea of parallelization, to calculate something that has no run-time order dependency in some area of a program on different nodes. When communication (such as broadcasting and reducing) with other nodes is needed, only one thread is selected to call MPI functions while other 7 threads wait until it finishes communication. This approach has less message load than if all threads in the process participate in MPI communication. So the slave nodes mainly do the computation work and will obey the order of the master node. It is common in a synchronization stage that sometimes a node has to wait for other nodes finishing calculating, in which the process of the program depends upon the slowest node, but data coherency is guaranteed under this framework.

Details of P-MIL are presented in Algorithm 1. *K* is the number of cancer subtypes, *T* is the number of iterations, *D* is the number of features and *N* is the number of compute nodes. In the line search algorithm, at the line 9 of Algorithm 1, [*left,right*] is the search interval, *ε* is precision limit and *B* is the number of search branches.



The process is designed to decrease the frequency of data scanning and MPI operations. In each inner iteration, we scan the whole data set only once when calculating the weighted histograms and scan the features for the best weak classifier once more to get $\mathbf {h}_{t}^{k}(x_{ij})$. The reductions of histograms for different features are merged into one MPI operation to save the time of synchronization among slaves, and it is similar while handling *loss*_1_,*loss*_2_,…,*loss*_*B*_.

## Results

In the experiments, we implement the parallel computing framework of P-MIL and apply it to large-scale high-resolution images.

For comparison purposes, MIL and MCIL are also parallelized and implemented in the experiments. Compared to P-MIL, the parallelized MCIL method has no max-margin concept among clusters to intensify the competition. Relative to the parallelized MCIL, the parallelized MIL method has no inner loop as well as latent variable. That is, no cluster classifier for each cluster is trained in the parallelized MIL. The boosting parts of the algorithms of these methods are alike. It is noteworthy that if the other two methods are not parallelized, their execution time is not comparable to that of P-MIL. By the way, ccMIL emphasizes on the segmentation task and uses contextual information that makes it difficult to implement a parallelized version of ccMIL, which is why ccMIL is not included in our experiments.

We verify the scalability of our framework and compare the accuracies of MIL, MCIL and P-MIL in image-level classification, pixel-level segmentation and patch-level clustering.

### Data set

We collect the image data set in the First Affiliated Hospital of Zhejiang University from May 1st to September 17th in 2011. The number of patients is 118. The number of the whole slices is 1318. The images are obtained from the Nano Zoomer 2.0-HT digital slice scanner produced by Hamamatsu Photonics with a magnification factor of 40. The study protocol was approved by the Research Ethics Committee of the Department of Pathology in Zhejiang University. All the individuals used for the analyses have provided written, informed consent.

We cut the images into pieces (each piece: 10,000×10,000 pixels) because the image size of 200,000×200,000 pixels is beyond the storage capacity of a single node. We randomly choose 13,838 pieces as the original training data set in our experiment (9868 cancerous and 3970 non-cancerous). The size of the original training data set is 3.78 TB. In the original training data set, each piece is labeled as cancer or non-cancer by two pathologists independently. If there exists a disagreement between two pathologists on a certain image, the two pathologists together with a third senior pathologist will discuss the result until a final agreement is reached. To evaluate the segmentation performance for testing purposes, we also choose 30 cancer pieces as testing data and label the corresponding cancerous regions. The testing data and training data are independent. The annotations also follow the above process to ensure the quality of labeled ground truth. It takes a total of 720 man-hours for three annotators to finish the labeling work. In addition, 30 cancer pieces, consisting of many instances, are representative, and we believe that they are reliable for testing.

For each piece, we extract patches using a step size of 100 pixels. For multi-scale, patches of three size-levels (160×160, 320×320 and 640×640) are extracted. 388,072,872 patches from 13,838 pieces are obtained.

A group of generic features are used for each patch, consisting of Color, Scale Invariant Feature Transform (SIFT) [61], Gray Level Histogram [62], Histogram of Oriented Gradient (HOG) [63], Locally Assembled Binary (LAB) [64], Gray Level Co-occurrence Matrix (GLCM) [65] and Region [66]. The SIFT algorithm captures interest points in an image as well as information about their scale and orientation to obtain local features. Even if the image is rotated, brightened or taken from different angles, the performance of the feature is still reliable. Cancer cells always have enlarged and hyper-chromatic nuclei, different from normal cells. By using the image gradient, SIFT descriptors are able to capture important features of objects, especially the appearances, thus able to distinguish cancer cells from normal cells. The Gray Level Histogram feature is statistics of the distribution of gray levels in an image, which shows information about the gray level frequency and the clarity of the image. The HOG feature uses the distribution of direction density of gradients or edges to build a good descriptor about the appearance and shape of an object. The LAB feature is a selectively reduced set of Assembling Binary Haar Features [64, 67]. By reduction, the LAB feature not only reduces the computation cost but also excels at face detection and other pattern recognition tasks. The GLCM feature captures texture information as well as structure information in an image. The Region feature shows higher discriminative power than single feature points in image matching because more representative information is extracted. The total feature dimension is 215. Due to the extremely large number of the patches, it takes 20 h in the feature extraction stage using eighty nodes.

We store our data set in an Redundant Arrays of Independent Disks 6 (RAID6) disk array, which can be accessed by every node. For readability and scalability, all the data is stored in plain-text format (ASCII code). In the data distribution stage, each node obtains the corresponding data, transforms them into binary format and saves the transformed data in local disk feature by feature, so that we can obtain high locality when we train a single-feature weak classifier. Furthermore, half of the RAM (8GB) in each node is used to cache the data set, as memory is orders of magnitudes faster than local disk. The data set is still in a disk array. What caching does here is it uses part of the internal memory as a sort of cache memory for faster access to data in the disk due to requirements for fast communication between nodes. In our experiments, we choose the Microsoft Windows HPC cluster as the platform. Nodes in the cluster are connected by network that enables low-latency, high-throughput application communication on the basis of Remote Direct Memory Access (RDMA) technology. Data blocks and messages are sent by using MPI implementations.

### Settings

The soft-max function we use here is the GM model and the weak classifier we use is multi-decision stump. For parameters, we set *K*=5, [*left,right*]=[0,1], *ε*=10^−5^ and *B*=100. The value of *T* varies on different experiments.

### Scalability

For parallel performance analysis, we carry out P-MIL on the large-scale data set with a varying number of nodes. We run 10 iterations because time used for each iteration is almost the same. Overall runtime, time of the data distribution stage, time of training the best weak classifier, time of searching the best alpha and the average amount of local disk storage used for each node are recorded in Table 1.

**Table 1:** Global statistics about time and storage for different numbers of nodes

N nodes	Cores	Overall[s]	Distribution[s]	Training[s]	Searching[s]	Storage[MB]
8	64	184361	22453	157183	4725	71384
16	128	157329	22352	132575	2402	31597
32	256	121897	20401	100214	1282	11709
64	512	21437	20485	313	639	1672
128	1024	21020	20491	203	326	0

The time for the data distribution stage heavily depends on the speed of disk array and the bandwidth of network. We use 64 network Input/Output (IO) threads for stability reasons in this stage for all experiments, which is why a varying number of nodes shared similar time in this stage. The training stage is data-intensive. In the experiments in which we are using 8, 16 and 32 nodes, RAM is not enough to keep the whole data set, so we have to scan data from local disk, which is time-consuming. When we are using 64 nodes, some data is stored in the local disk, notwithstanding the operating system would automatically load them into the kernel buffer. So in the experiments of 64 and 128 nodes, all the data could be loaded into memory, in which we could indeed achieve high computing performance. Due to the inherence of the searching algorithm as well as the size of the data, the searching stage is inevitably compute-intensive. The searching algorithm we choose is brute-force but highly parallel, so this stage could speed up almost linearly.

In Table 2, we show the communication, synchronization and computation time of the training and searching stages using 64 and 128 nodes. Comm.&sync. time makes up of communication time and synchronization time among compute nodes. An example of synchronization is that, in the best $\alpha _{t}^{k}$ searching stage, the master has to get summarization of all the *loss*_*i*_ values to determine the next search interval to search, so the slaves which finish calculating *loss*_*i*_ faster have to wait for the slower ones. Communication time is the sum of the time costs of calling MPI functions. To measure the load balance, we report the average time, the maximum time and the ratio between each pair of each part. The closer this ratio is to 1, the better the load balance.

**Table 2:** Load balance statistics about training and searching time for 64 and 128 nodes

Stage	Comm. & Sync.			Computation		
	avg.[s]	max.[s]	avg./max.	avg.[s]	max.[s]	avg./max.
64-training	58.1	64.1	0.91	254.5	310.0	0.82
64-searching	275.4	294.4	0.94	363.7	589.9	0.62
128-training	81.4	90.9	0.90	121.4	200.2	0.61
128-searching	147.3	160.6	0.92	178.8	324.8	0.55

In a word, in each iteration, it takes us only 53 s to process 388 million instances with 128 nodes. In Tables 1 and 2, it is indicated that our framework is available with even fewer nodes, which also shows that the parallelization strategy is both efficient and effective.

### Classification, segmentation and clustering

**Fig. 4:**
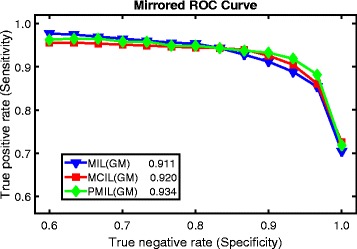
The mirrored Receiver Operating Characteristic (ROC) curve for comparisons of piece-level classification results with Multiple Instance Learning (MIL), Multiple Clustered Instance Learning (MCIL) and Parallel Multiple Instance Learning (P-MIL). The generalized mean (GM) model is the soft-max function in the methods

For accuracy analysis, we use the parallelized MIL, the parallelized MCIL and P-MIL to make comparisons, based on the MIL-Boost framework. We follow the standard leave-one-out method in a six-fold cross validation. 128 nodes are used to train MIL, MCIL and P-MIL models on the training data with *T*=500 iterations using the same features and parameters. It takes 3.2 h (500 iterations), 11.5 h (500 iterations and *K*=5) and 11.6 h (500 iterations and *K*=5) for MIL, MCIL and P-MIL respectively in a training process. If these methods are not parallelized, with the core number we used changed to one, it will take about 4.2 months, 15.2 months and 15.3 months for MIL, MCIL and P-MIL respectively to run in a training process. We can see how much time will MIL and MCIL take to process on a single core if not parallelized, which is not comparable to P-MIL at all in processing time. The parallelized MIL has no clustering in its program so its processing time is obviously smaller. With a competition concept introduced to enhance accuracy, P-MIL is expected to take slightly more time than the parallelized MCIL does. The three models are tested on the test data for piece classification (cancer vs non-cancer). Figure 4 shows the Receiver Operating Characteristic (ROC) curves [68] with regard to the classification results. As is shown in Fig. 4, the F-measure of P-MIL is slightly better than the other two methods. So P-MIL is not only much faster than the other two methods, but also has higher classification accuracy.

The 30 cancer pieces are used to evaluate segmentation results. The F-measure is used for the quantitative measurement of segmentations. The F-measure values are 28.2*%*, 43.1*%* and 61.7*%* for MIL, MCIL and P-MIL respectively in 30 labeled positive images. The P-MIL significantly improves segmentation results by increasing competition among clusters. MIL-based approaches are a form of weakly supervised learning, closely related to semi-supervised machine learning. According to [69], an F-measure value of 60% in segmentation results is already relatively high in weakly supervised learning. Furthermore, our segmentation results are mainly to help pathologists to locate the cancerous regions. A partial cancer region suggested by the segmentation is enough to guide the pathologists to search for more detailed cancer regions.

**Fig. 5:**
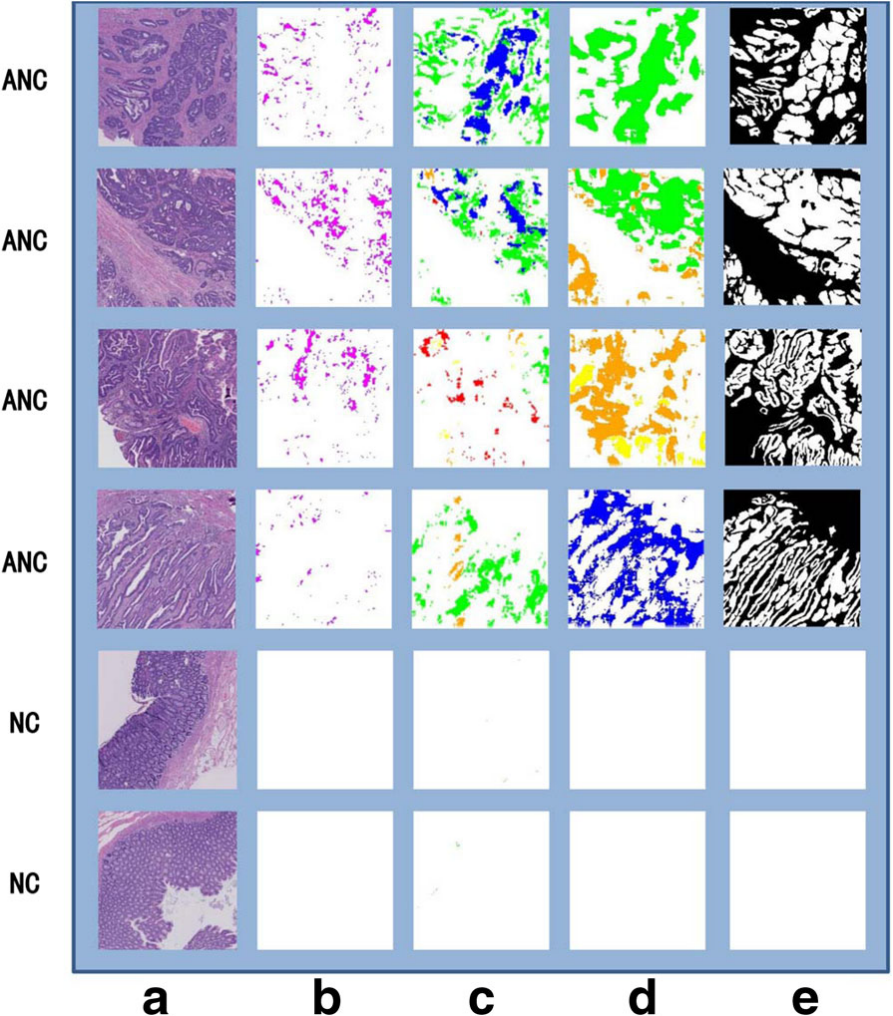
Image Types: **a**: The original images. **b**–**d**: The instance-level segmentations for MIL, MCIL and P-MIL respectively. **e**: The ground truth. ANC: abnormal; NC: normal. Different colors represent different cancer subtypes

Since we mainly focus on clustering performance here, we only include true positive instances as the measured data. In the clustering evaluation measure stage, purity is chosen as external criteria for evaluating how well clustering results fits in standard answers. The purity of P-MIL is 99.89*%* while the purities of MIL and MCIL are 97.3*%* and 98.1*%* respectively (*K*=5). Due to the max-margin concept among different clusters, the purity performance of P-MIL is better than those of the other two methods. The achieved purity is almost 100.0*%*, which is challenging to achieve. As shown in Fig. 5, the P-MIL algorithm is both effective and efficient.

## Discussion

P-MIL outperforms MIL and MCIL in execution time, image-level classification, pixel-level segmentation and patch-level clustering according to previous analysis and Fig. 4. In addition, P-MIL is a general but effective and efficient method, can be performed in real-time.

As expected, the results show that the searching and training efficiency increases almost linearly as the number of nodes increase. The time of distributing data obviously cannot be reduced by increasing nodes but the training time is reduced sharply in this way. In consideration of cost, there might be a trade-off point to balance the cost of the platform and the efficiency of the program. For instance, an optimal ratio of performance to cost can be calculated and chosen by the user. Moreover, since HPC Cloud Computing is available, in which we can easily request the number of nodes we require, we do not need plenty of nodes to participate in computing if there is only a small amount of training data to train and predict.

P-MIL shows high accuracy for the test data in evaluation. Its main difference with MIL and MCIL in the learning stage is the introduction of competition. Enhancing competition among clusters and updating the new clusters indeed contribute to the high accuracy of the test results.

In clustering, we set the number of clusters, K, to a value greater than four to explore new possible cancer subtypes. There are four major subtypes of colon cancer, including: moderately or well differentiated tubular adenocarcinoma, poorly differentiated tubular adenocarcinoma, mucinous adenocarcinoma and signet-ring cell carcinoma [70]. Different visual patterns in clusters may divide a known cancer pattern into cancer subtypes. Since there exists no clear standard or definition for subclasses and subtypes, our method is potentially helpful for discovering new cancer subtypes in classification. In the future, we will conduct experiments with different values of K and cooperate with pathologists to further validate the clustering results in clinical practice.

## Conclusions

We propose the P-MIL algorithm for large scale histopathology image analysis using the MPI/multi-threading programming model on the Microsoft HPC cluster to perform classification, segmentation and clustering altogether. The concept of max-margin is introduced to the P-MIL framework to improve clustering results. As a result of max-margin, our method achieved high performance in the aforementioned tasks. The parallel framework significantly shortens the execution time, which makes our method viable in practical application. At last, we successfully complete the experiment using extremely large histopathology images in 128 nodes for 11.6 h. Our results demonstrate that P-MIL scales up almost linearly and achieves satisfactory performance, including classification, on a large-scale data set up to a size of 3.78 TB. The short prediction time of about 382.79 s for one patient’s images suggests the clinical application value of our method. Our experiments show that we could apply the method to efficient histopathology image analysis, through which, if possible, a cancerous patient’s prognosis could be almost accurately made. One advantage of choosing HPC as the parallel computing platform is that Windows Azure added support for HPC and MPI recently, which enables our algorithm to scale up to cloud with minor porting effort.

Furthermore, this work can also be extended to achieve better performance and results. First, as a general model, P-MIL can be applied to other image types in addition to colon cancer images. We are currently collecting a complete data set of large-scale brain tumor images for further testing of our algorithm. Moreover, the boosting framework in our method could be replaced with other types of boosting, which would probably improve the performance. In addition to max-margin, there might be some other ways to enhance the competition among clusters. Besides, the parallel model could be further improved. For example, we could adopt a more strongly parallel hybrid model to enhance the degree of parallelization and running efficiency. Time complexity and space complexity of our algorithm may still have room for improvement. In the near future, we will migrate P-MIL from HPC to Azure to benefit from the larger computing power.

## Abbreviations

CAD: Computer aided diagnosis
CBIR: Content based image retrieval
ccMIL: Context-constrained multiple instance learning
GLCM: Gray level co-occurence matrix
GM: Generalized mean
HOG: Histogram of oriented gradient
HPC: High-performance-computing
IO: Input/Output
KNN: k-Nearest neighbor
LAB: Locally assembled binary
LSA: Latent semantic analysis
MCIL: Multiple clustered instance learning
MIL: Multiple instance learning
MIP: Maximum intensity projection
MIL-Boost: Multiple instance learning-boost
MPI: Message passing interface
OpenMP: Open multi-processing
PC: Personal computer
PMIL: Parallel multiple instance learning
RAM: Random access memory
RAID6: Redundant arrays of independent disk 6
RDMA: Remote direct memory access
ROC: Receiver operating characteristic
SIFT: Scale invariant feature transform
SVMs: Support vector machines
